# A novel *NONO* variant that causes developmental delay and cardiac phenotypes

**DOI:** 10.1038/s41598-023-27770-6

**Published:** 2023-01-18

**Authors:** Toshiyuki Itai, Atsushi Sugie, Yohei Nitta, Ryuto Maki, Takashi Suzuki, Yoichi Shinkai, Yoshihiro Watanabe, Yusuke Nakano, Kazushi Ichikawa, Nobuhiko Okamoto, Yasuhiro Utsuno, Eriko Koshimizu, Atsushi Fujita, Kohei Hamanaka, Yuri Uchiyama, Naomi Tsuchida, Noriko Miyake, Kazuharu Misawa, Takeshi Mizuguchi, Satoko Miyatake, Naomichi Matsumoto

**Affiliations:** 1 Department of Human Genetics, Yokohama City University Graduate School of Medicine, 3-9 Fukuura, Kanazawa-Ku, Yokohama, Kanagawa Japan; 2 Brain Research Institute, Niigata University, Niigata, Japan; 3 School of Life Science and Technology, Tokyo Institute of Technology, Yokohama, Kanagawa Japan; 4 Biomedical Research Institute, National Institute of Advanced Industrial Science and Technology (AIST), Tsukuba, Ibaraki Japan; 5 Children’s Medical Center, Yokohama City University Medical Center, Yokohama, Kanagawa Japan; 6 Department of Pediatric Cardiology, Yokohama City University Hospital, Yokohama, Kanagawa Japan; 7 Department of Pediatrics, Fujisawa City Hospital, Fujisawa, Kanagawa Japan; 8 Department of Medical Genetics, Osaka Women’s and Children’s Hospital, Izumi, Osaka Japan; 9 Department of Rare Disease Genomics, Yokohama City University Hospital, Yokohama, Kanagawa Japan; 10 Department of Human Genetics, Research Institute, National Center for Global Health and Medicine, Tokyo, Japan; 11 RIKEN Center for Advanced Intelligence Project, Tokyo, Japan; 12 Clinical Genetics Department, Yokohama City University Hospital, Yokohama, Kanagawa Japan

**Keywords:** Clinical genetics, Medical genetics, Neurodevelopmental disorders

## Abstract

The *Drosophila* behavior/human splicing protein family is involved in numerous steps of gene regulation. In humans, this family consists of three proteins: SFPQ, PSPC1, and NONO. Hemizygous loss-of-function (LoF) variants in *NONO* cause a developmental delay with several complications (e.g., distinctive facial features, cardiac symptoms, and skeletal symptoms) in an X-linked recessive manner. Most of the reported variants have been LoF variants, and two missense variants have been reported as likely deleterious but with no functional validation. We report three individuals from two families harboring an identical missense variant that is located in the nuclear localization signal, *NONO*: NM_001145408.2:c.1375C > G p.(Pro459Ala). All of them were male and the variant was inherited from their asymptomatic mothers. Individual 1 was diagnosed with developmental delay and cardiac phenotypes (ventricular tachycardia and dilated cardiomyopathy), which overlapped with the features of reported individuals having *NONO* LoF variants. Individuals 2 and 3 were monozygotic twins. Unlike in Individual 1, developmental delay with autistic features was the only symptom found in them. A fly experiment and cell localization experiment showed that the *NONO* variant impaired its proper intranuclear localization, leading to mild LoF. Our findings suggest that deleterious *NONO* missense variants should be taken into consideration when whole-exome sequencing is performed on male individuals with developmental delay with or without cardiac symptoms.

## Introduction

The *Drosophila* behavior/human splicing (DBHS) protein family is involved in numerous steps of gene regulation, including transcriptional regulation, RNA processing and transport, and DNA repair. The DBHS proteins are characterized by the domains of N-terminal RNA recognition motifs (RRMs), a NonA/paraspeckle domain (NOPS), and a C-terminal coiled-coil domain. A nuclear localization signal (NLS) is located at their C-terminus. Three members of the DBHS protein family are found in humans: SFPQ (splicing factor, proline- and glutamine-rich: MIM 605199), PSPC1 (paraspeckle component 1: MIM 612408), and NONO (non-pou domain-containing octamer-binding protein: MIM 300084)^1^.

*NONO* is located at chromosome Xq13.1, and its hemizygous loss-of-function (LoF) variants cause X-linked syndromic intellectual developmental disorder-34 (MRXS34: MIM 300967). Clinical features of MRXS34 include delayed psychomotor development, intellectual disability, slender build, dysmorphic features, heart defects (e.g., left ventricular noncompaction, atrial septal defect, etc.), and other multiple-organ manifestations such as in skeletal or muscle/soft tissues. Several individuals with *NONO* variants have been reported; most of these variants were LoF variants and no missense variants in *NONO* associated with human phenotypes have been reported^2–9^. Two missense variants have been reported, but with no functional evaluation^10,11^. Therefore, it is still unknown that how missense variants in *NONO* cause human diseases.

Here, we report three individuals harboring an identical missense variant in *NONO*, NM_001145408.2:c.1375C > G p.(Pro459Ala), which is located in the NLS. Some of their clinical features overlapped with the symptoms caused by LoF variants in *NONO*. Functional study by fly and cell localization experiments disclosed the nature of this variant.

## Materials and methods

### Editorial policies and ethical considerations

Clinical information was obtained from corresponding doctors and medical records. Informed consent was obtained from the guardians of all individuals. The experimental protocols were approved by the Committees for Ethical Issues of Yokohama City University Faculty of Medicine (Yokohama, Japan). All procedures involving human subjects were performed in accordance with the Declaration of Helsinki.

### Subjects

Three individuals from two families were included in this study. Individual 1’s main clinical symptoms were febrile seizures, mental retardation, and dilated cardiomyopathy. Individuals 2 and 3, who were monozygotic twins, were diagnosed with developmental delay and autism.

### Genetic analysis

Genomic DNA was obtained from peripheral blood leukocytes of all individuals using QuickGene 610L (Wako, Osaka, Japan). We performed whole-exome sequencing (WES) as previously described^12^. In brief, genomic DNA was captured using SureSelect Human All Exon V5 or V6 (Agilent Technologies, Santa Clara, CA, USA), and the captured libraries were sequenced on an Illumina HiSeq 2500 with 101-bp paired-end reads. Image analysis and base calling were performed using sequence control software with real-time analysis and CASAVA software (Illumina, San Diego, CA, USA). Sequence reads were aligned to GRCh37 using Novoalign (http://www.novocraft.com/). Local realignments around indels and base quality score recalibration were performed using Picard (http://picard.sourceforge.net/) and the Genome Analysis Toolkit (GATK; https://www.broadinstitute.org/gatk/index.php). Variants were called by GATK UnifiedGenotyper and filtered according to GATK Best Practices V3 (https://software.broadinstitute.org/gatk/). Included variants were annotated using ANNOVAR software (http://annovar.openbioinformatics.org/) after excluding common variants registered in the common dbSNP135 data (minor allele frequency > 0.01). The detected variants were confirmed by Sanger sequencing.

### Variant prioritization

Trio-based WES was performed on the two families. After filtering variants by minor allele frequency according to each manner of inheritance, we manually assessed each variant by focusing on gene constraint metrics^13^, expression patterns, and prediction using the in silico tools SIFT^14^, PolyPhen-2^15^, MutationTaster^16^, and CADD^17^.

### Fly strains

Flies were maintained at 25 °C on standard fly food. The fly strains *GMR-Gal4* (#1104), *Tub-Gal80*^*ts*^ (Bloomington 7019), *Tub-Gal4* (Bloomington 5138), and *c739-Gal4,UAS-mCD8::GFP* (#64305) were obtained from the Bloomington Stock Center, while *40D-UAS* (#60101) for the control experiment was purchased from VDRC (Vienna, Austria).

### Generation of the fly line for expression of NONO variants and fly ortholog

To express NONO wild type and the Pro459Ala variant in *Drosophila*, NONO cDNA was amplified by PCR from the cDNA library of human cortical neural stem cells. NONO Pro459Ala was introduced by PCR-mediated site-directed mutagenesis using PrimeSTAR Max DNA Polymerase (Takara, Shiga, Japan). The Myc tag was fused to the N-terminus of the wild type (WT) or the Pro459Ala mutation, and they were inserted into the vector pUASTattB (Vectorbuilder). These plasmids were then injected into y[1] M{vas-int. Dm}ZH-2A w[*]; PBac{y[+]-attP-3B}VK00037 embryos for insertion into the *VK00037* landing site using site-specific integration via the phi-C31 system (WellGenetics, New Taipei, Taiwan).

For the fly homologous gene *nonA* (*dNONO*), its cDNA (RE 58280) was obtained from the Drosophila Genomics Resource Center (Indiana University). This was inserted into the pUASTattB vector. Then, it was inserted into the landing site of VK00037 to produce a transgenic line as well as NONO.

### Western blot analysis

Protein expression was temporarily induced throughout the body of *Drosophila* using expression systems combining Tub-Gal4 and Tub-Gal80^ts^. The flies produced by crossing NONO WT and Pro459Ala were raised at a permissive temperature (20 °C), and newly eclosed females were collected from vials and held at a restrictive temperature (29 °C). After 1 day, five flies were homogenized using a BioMasher II (Nippi, Tokyo, Japan), and the homogenates were lysed in lysis buffer (10 mM Tris–HCl, pH 7.5, 150 mM NaCl, 1 mM EDTA, 2% DDM) supplemented with protease inhibitor cocktail (Protease Inhibitor Cocktail Set III; Calbiochem, La Jolla, CA, USA). The following reagents were used for western blotting: mouse anti-myc (4A6) (1:5,000; 05-724-25UG; EMD Millipore Corp., USA) and mouse anti-α-tubulin (T9026, 1:10,000; Sigma‐Aldrich, St. Louis, MO, USA) antibodies, iBlot2 NC Stacks nitrocellulose membranes (Thermo Fisher Scientific), and skimmed milk. For signal detection, we used Amersham ECL Prime Western Blotting Detection Reagents (Cytiva, Marlborough, MA, USA) as per the manufacturer’s instructions. Images were captured using MultiImager II ChemiBox (BioTools Inc., Japan).

### Eye imaging using bright-field microscopy and the quantification of morphological defects in the eye

For light microscope imaging of adult eyes, 1-day-old flies, reared at 20 °C for the expression of NONO WT and NONO Pro459Ala, and at 29 °C for the expression of dNONO by GMR-Gal4, were immobilized by freezing at − 80 °C, after which the flies were mounted on a microscope slide with double-sided tape. These fly eyes were imaged using a BX53 microscope system with an MPLFLN 20× objective lens (Olympus, Tokyo, Japan). The phenotypic scores were calculated using Flynotyper(Iyer et al., *G3*, 2016). Experimental analyses were performed using Prism 9 (GraphPad Software Inc., San Diego, CA, USA). The distribution of our data was determined using the D’Agostino & Pearson test and the Kolmogorov–Smirnov test (the normality test was passed if *P* < 0.05). For data following a Gaussian distribution, we used ordinary one-way ANOVA with Tukey’s multiple comparisons between groups.

### Immunostaining and imaging

Experimental procedures for brain dissection, fixation, and immunostaining were as previously described(Sugie et al., J. Vis. Exp., 2017). Briefly, the brain was dissected in PBS, fixed in 4% formaldehyde (Electron Microscopy Sciences, USA) for 50 min at room temperature, and washed three times with 0.3% PBT; then, mouse anti-myc (9B11, 2276S; Cell Signaling Technology Inc., USA) was diluted 400-fold in 0.3% PBT and incubated overnight at 4 °C in the solution. After three washes with 0.3% PBT, the secondary antibody was incubated with mouse Alexa 568 (A32723, ThermoFisher) at 400-fold dilution and with PureBlu DAPI Nuclear Staining Dye (#1351303; Bio-Rad, USA) at 100-fold dilution and 4 °C overnight. Finally, it was washed three times with 0.3% PBT, mounted with Vectorshield (H-1000; Funakoshi, Japan), and scanned with a confocal microscope (FV3000; Olympus). The obtained data were 3D-constructed using Imaris image analysis software (Bitplane).

### Quantification of diffused NONO localization

The surface function of IMARIS image analysis software was used to extract only the region where the myc signal intensity of NONO was high (I). Next, the region containing the weak myc signal of NONO was extracted (II). Each was then expressed numerically as a volume (μm^3^). We then calculated the diffused NONO signals by subtracting (I) from (II). The quantification was performed by an experimenter who was blind to the genotype.

## Results

### Case report

Table 1 summarizes the clinical features of Individuals 1–3. All of them had intellectual disability and developmental delay. Individual 1 had cardiac symptoms. Unlike previous reports on cases with *NONO* variants, none of these individuals had distinctive facial features, motor/neurological findings, or skeletal abnormalities.

**Table 1:** Clinical features of three individuals with a missense variant in *NONO.*

	Family 1	Family 2	
	Individual 1	Individual 2	Individual 3
Inheritance	Maternal	Maternal	Maternal
Nucleotide change	c.1375C > G	c.1375C > G	c.1375C > G
Amino acid change	p.(Pro459Ala)	p.(Pro459Ala)	p.(Pro459Ala)
Sex	Male	Male	Male
Age at examination	2y2m	4y5m	4y5m
Current Age	6y6m	10y	10y
Developmental milestone			
Motor development			
Head control	3-4 m	NA*	NA*
Rolling over	NA*	NA*	NA*
Crawling	NA*	NA*	NA*
Sitting independently	10 m	NA*	NA*
Walking without assistance	1y9m	15m	15m
Intellectual development			
Eye tracking	NA*	NA*	NA*
Social smile	NA*	NA*	NA*
Use of a single meaningful word	4y6m	Not acquired	Not acquired
Two-word sentence	4-5y	Not acquired	Not acquired
Regression	−	−	−
Intellectual disability	+	+	+
Epilepsy	+	−	−
Abnormal behaviors	+	+	+
Dysmorphology	−	−	−
Craniofacial/Neck features	−	−	−
Chest			
Cardiac defects	+	−	−
Others	−	−	−
Abdominal/Pelvic features	−	−	−
Motor/Neurological findings			
Abnormal muscular tonus	−	−	−
Feeding difficulties	−	−	−
Involuntary movements	−	−	−
Ataxia	−	−	−
Others	−	−	−
Skeletal abnormalities			
Scoliosis	−	−	−
Abnormal findings in hand/foot	−	−	−
Other symptoms/findings	−	−	−
Head MRI findings	−	Not performed	Not performed

*Acquired but specific periods were not obtained.

Individual 1 was an only male child born to non-consanguineous Japanese parents. A nephew on his father’s side had a history of complex febrile seizures. He was conceived by intracytoplasmic sperm injection. The mother suffered from gestational diabetes mellitus that was under good control with intake restriction. He was born by normal vaginal delivery at 39 weeks and 4 days; his birth weight was 3575 g, length was 50.2 cm, head circumference was 34.5 cm, and Apgar scores at 1 and 5 min were 8 and 9, respectively. At 4 months, he suffered a bout of vomiting and was transferred to an emergency room, where he was diagnosed with pulseless ventricular tachycardia (VT) attacks, while subsequent investigation diagnosed him with idiopathic dilated cardiomyopathy. He was hospitalized for 12 months to treat the VT attacks until the age of 1 year and 6 months old. His VT attacks continued intermittently for 6 months until 10 months old. Multiple antiarrhythmic drugs were administered for the refractory VT attacks. He took amiodarone, mexiletine, and sotalol at the time of discharge. At 6 months, he had a generalized tonic–clonic seizure attack during a VT attack. At 11 months, he had status epilepticus following a fever. At 1 year and 4 months, he again had a tonic seizure with loss of consciousness and eyeballs rolling upwards. He was diagnosed with epilepsy by an electroencephalography test at 1 year and 5 months, at which time he started levetiracetam, although it was not effective. After having tried several medications, the combination of clonazepam and valproic acid controlled his seizures (the last attack was at 3 years and 5 months). The epilepsy medication was then successfully tapered and treatment with clonazepam alone has been performed since the age of 6 years. On his last hospital visit at 6 years and 6 months, his cardiac function had not worsened (left ventricular ejection fraction: 45%) and he was able to perform normal daily activities. He had developmental delay and intellectual disability, with an intellectual capacity equivalent to that of a 3- to 4-year-old child. He could communicate with others by speaking up to three-word sentences. Although his socialization and group adaptability appeared to have improved, he also showed hyperactivity and impulsivity.

Individuals 2 and 3 were monozygotic twins born to non-consanguineous Japanese parents. Their mother had no complications during her pregnancy and delivery. Individual 2 was the older twin born at 37 weeks of gestation with a birth weight of 2710 g. He had intussusception that was treated by reduction at 1 year and febrile seizure following roseola infantum at 2 years. He had not suffered afebrile convulsion or seizure. Although he had acquired normal motor development, his language expression was markedly delayed and he could not speak any meaningful words. He also could not urinate by himself or express the urge to urinate, so he used diapers. He was prone to injuring himself, such as hitting his head. When he was bothered by noise in the surroundings, he covered his ears and occasionally exhibited panicked behavior. Individual 3 was the younger twin with a birth weight of 2498 g. He did not have any remarkable past medical history. Similar to Individual 2, his motor development was normal, but he had severe intellectual disability with autistic features. He had not acquired the ability to speak any meaningful words. He was prone to injuring himself, such as by hitting his head. He was also sensitive to noise in the surroundings, which he attempted to block out by covering his ears. He could not urinate by himself or express the urge to urinate, and thus he used diapers. He also had poor perception of risk.

### Genetic analysis

**Figure 1:**
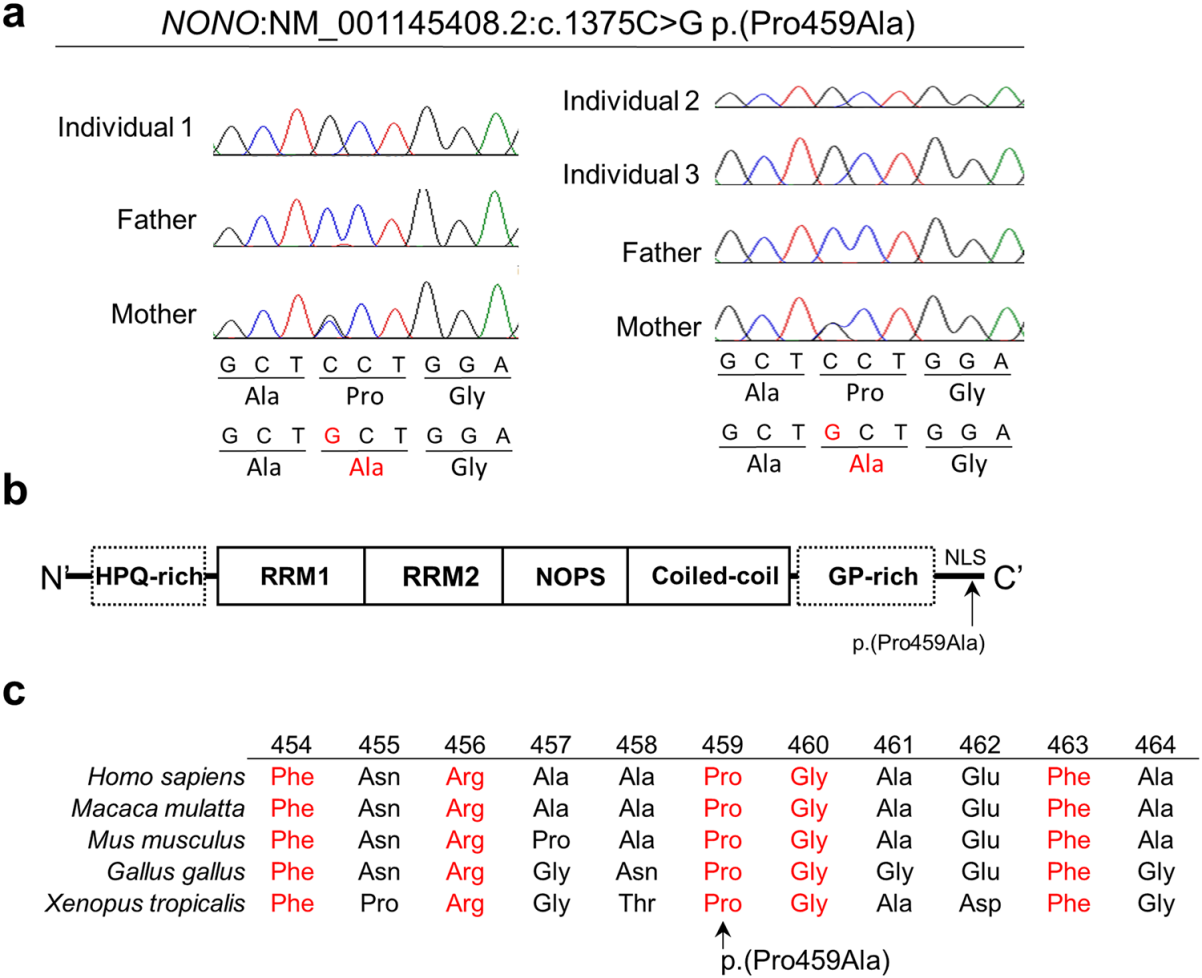
Genetic analysis of *NONO* variant. (**a**) Electropherograms show that Individuals 1–3 had a hemizygous *NONO* variant inherited from their mother. The altered nucleotide and amino acid are shown in red. (**b**) Schematic presentation of NONO domains (solid line) and regions (dashed line), and the identified variant (arrow). NLS, nuclear localization signal; NOPS, NonA/paraspeckle; RRM, RNA recognition motif. (**c**) Evolutionary conservation of NONO protein and its orthologs within vertebrates. Conserved amino acids are shown in red.

Trio-based WES was performed and identified an identical hemizygous variant in *NONO*: c.1375C > G p.(Pro459Ala), in Individuals 1–3. Sanger sequencing confirmed that the *NONO* variant had been inherited from their asymptomatic mother (Fig. 1a). This variant was located at a highly conserved NLS region (Fig. 1b) and was absent from our in-house and public control databases (esp6500 and gnomAD). In silico predictions supported its pathogenicity (SIFT: 0.018, PolyPhen 2-HVAR: 0.956, MutationTaster score/prediction: 1/disease causing, and CADD phred: 20.5). In addition to this variant, we did not identify any other variants that are known to cause the patients’ symptoms. Because it was previously shown that *NONO* LoF variants increased the levels of expression of other proteins in the DBHS protein family (SFPQ and PSPC1)^2^, we suspected that this variant inhibited the proper function of *NONO*. We performed western blot analysis to evaluate the expression levels of NONO, SFPQ, and PSPC1 using Individual 1’s lymphoblastoid cell line. Compared with the levels in the normal control, the expression levels of SFPQ and PSPC1 were unchanged (data not shown), necessitating a more detailed functional assessment.

### Evaluation of the pathological significance of *NONO* variant in *Drosophila*

**Figure 2:**
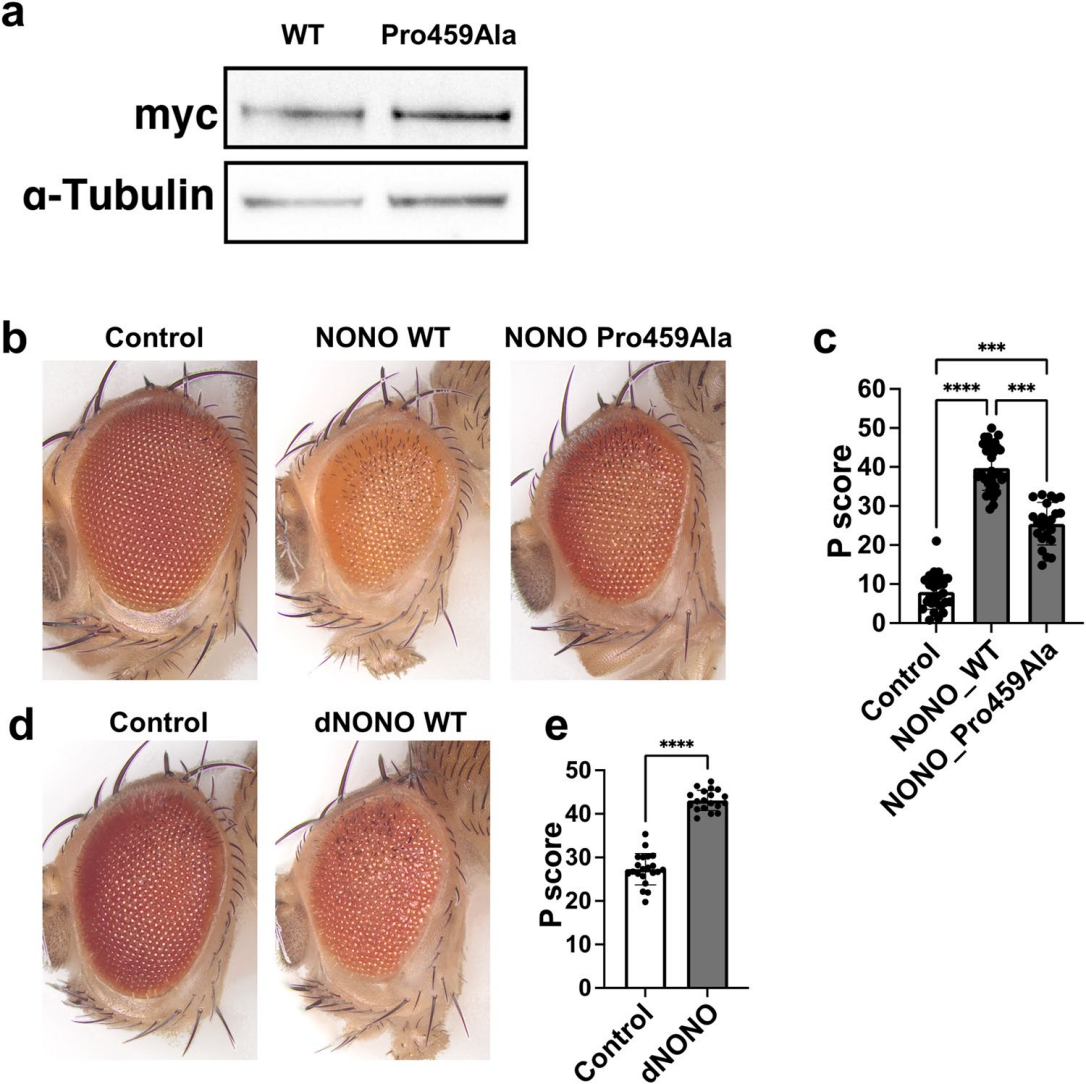
Functional evaluation of NONO variant in *Drosophila.* (**a**) Western blotting confirmed the expression of the NONO WT and the Pro459Ala variant. Myc-NONO WT or Myc-NONO Pro459Ala was expressed throughout *Drosophila* bodies and detected with anti-Myc antibody. Anti-α-tubulin was used as a loading control where indicated. (**b**) Representative bright-field microscope images of fly eyes displaying eye-specific ectopic expression of Myc-NONO WT and Myc-NONO Pro459Ala using the GMR-Gal4 driver, reared at 20 °C. (**c**) Quantification results of the phenotypic scores in control (*n* = 29), Myc-NONO WT (*n* = 32), and Myc-NONO Pro459Ala (*n* = 23). Data represent the mean ± SD. Statistical comparisons were conducted using nonparametric ANOVA (Kruskal–Wallis test) followed by Dunn’s multiple comparisons test. n.s.: not significant. ***P < 0.001, ****P < 0.0001. (**d**) The fly eyes displaying eye-specific overexpression of *nonA* using the GMR-Gal4 driver, reared at 29 °C. (**e**) Quantification results of the phenotypic scores in control (*n* = 21) and *nonA* (*n* = 19). Data represent the mean ± SD. Statistical comparisons were conducted using parametric test (unpaired t test with Welch’s correction). n.s.: not significant. ****P < 0.0001.

To evaluate the effects of the p.Pro459Ala mutation in *NONO *in vivo, we generated transgenic flies to express wild-type or Pro459Ala mutations of human NONO in a tissue-specific manner. The myc tag was fused upstream of each *NONO* gene to assess NONO protein expression and localization patterns. The *NONO* WT and Pro459Ala were inserted into the same position in the genome using the phiC31 integrase system(Groth et al., Genetics 2004), and thus no positional effect of gene expression was expected. Each variant was then expressed throughout the body, and production of the NONO protein was confirmed by western blotting. Both wild-type and mutant NONO proteins were detected at the expected size (55 kDa) (Fig. 2a).

Next, we expressed each variant in *Drosophila* eye using glass multiple reporter (GMR)-Gal4(Newsome et al., Cell 2000) to compare the function of the wild-type NONO and the mutant variant at 20 °C. The expression of wild-type NONO had an effect on the retina and showed the disordered alignment and dysmorphic structure of compound eyes (Fig. 2b, c). Pro459Ala had significantly milder phenotypic scores regarding structural abnormality (Fig. 2b, c). Note that human NONO wild-type expression was reported to be lethal at 25 °C because increased temperature was shown to cause increased expression of GAL4 (Kramer and Staveley, Genet. Mol. Res, 2003), leading to higher expression of NONO.

Notably, overexpression of the *Drosophila* ortholog *nonA* (*dNONO*) in the fly eye at 29 °C resulted in structural abnormalities of the retina similar to those in human NONO. This implies that the gene functions of *dNONO* and *NONO* are conserved (Fig. 2d, e). Together with these results, it was suggested that Pro459Ala is a possible loss-of-function mutation, resulting in a weakened rough-eye phenotype.

**Figure 3:**
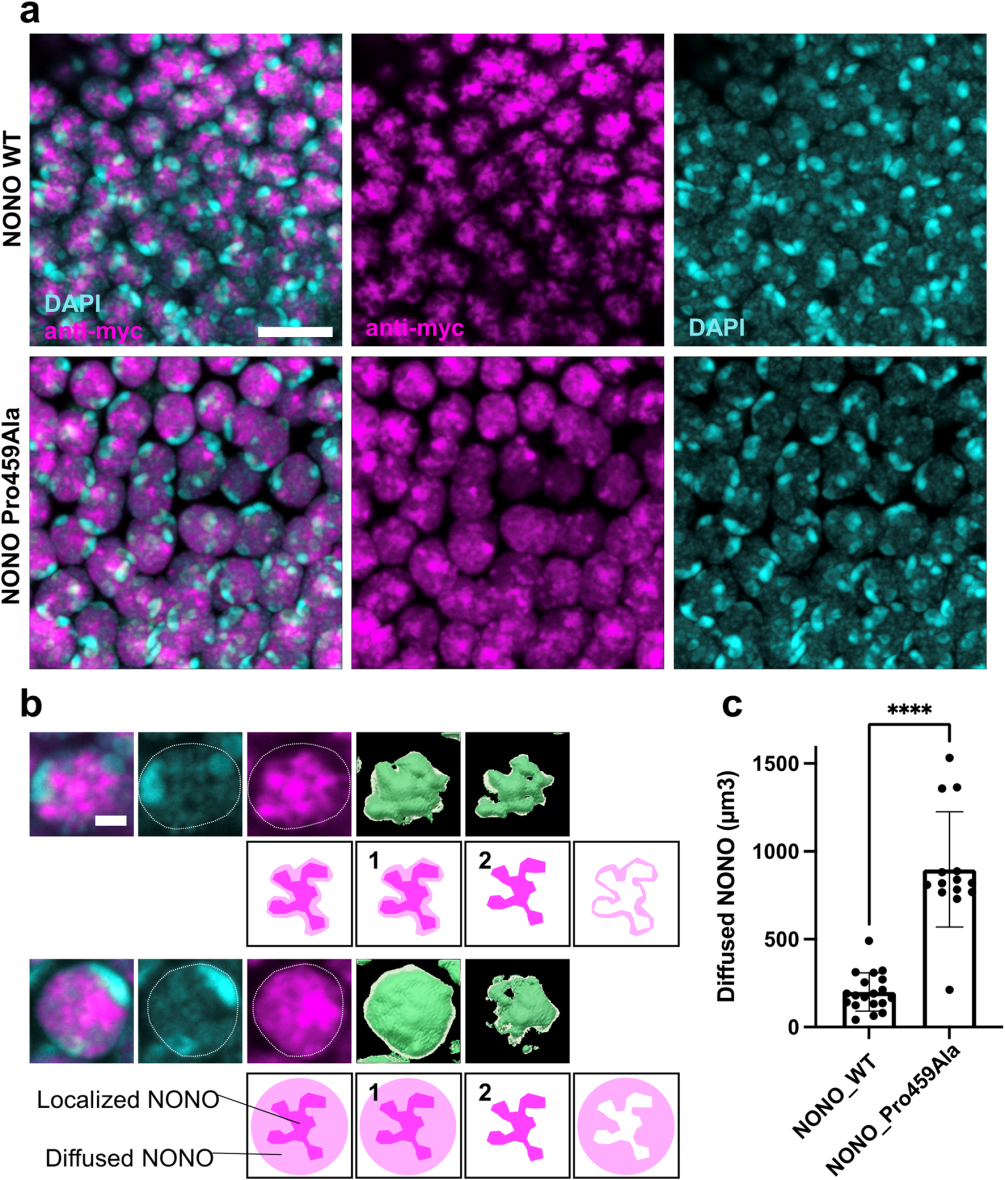
Localization pattern of NONO variant in *Drosophila* neuron. (**a**) Cell bodies of Kenyon cells in *Drosophila* brain. Wild-type NONO and Pro459Ala NONO are expressed. The localization pattern of Myc-NONO (magenta) was stained with anti-Myc antibody using immunohistochemistry. The nuclei were visualized using DAPI (cyan). Scale bar = 5 µm. (**b**) Comparative localization patterns of NONO variants in a Kenyon cell. Myc-NONO is shown by anti-myc antibody and the nucleus is visualized using DAPI. NONO is strongly localized in the weak region of DAPI. The Imaris image analysis software extracted (1) Myc signals, including the strong and weak signals, and (2) only strong Myc signals. They were then converted into numerical form as volumes (µm^3^). Diffuse signals in the nucleus were then calculated by subtracting (2) from (1). Scale bar = 1 µm. (**c**) Quantification results of the diffuse signals in the nucleus (µm^3^) in Myc-NONO WT (*n* = 19) and Myc-NONO Pro459Ala (*n* = 14). Data represent the mean ± SD. Statistical comparisons were conducted using a nonparametric Mann–Whitney test. ***P < 0.0001.

Next, we compared the subcellular localization patterns of wild-type and mutant NONO. To perform this analysis, Myc-NONO was expressed in a specific type of neuron called Kenyon cells with a *c739-Gal4* driver(Yang et al., Neuron 1995). Both the wild-type and mutant forms of NONO were located in the nucleus (Fig. 3a). The wild type of NONO was strongly localized in the nucleus where the signal of nuclear marker DAPI was weak. Compared with the findings in the WT, Pro459Ala showed diffuse signals in the nucleus (Fig. 3b, c). This suggests that Pro459Ala slightly weakens the function localized at the specific site in the nucleus.

## Discussion

We identified a recurrent hemizygous *NONO* missense variant in three individuals with neurodevelopmental disorder and cardiac symptoms. Fly experiments showed that this variant impeded proper intranuclear localization, leading to mild LoF. Most of the reported *NONO* variants are LoF variants^2–9^, and no functional validation for reported missense variants in *NONO*^10,11^. Our results suggest that deleterious *NONO* missense variants cause similar phenotypes by disrupting proper nuclear localization, leading to mild LoF.

The *NONO* variant, c.1375C > G p. (Pro459Ala), was identified in three male individuals with various symptoms. Individual 1 had intellectual disability and cardiac symptoms, which were consistent with the findings in other reported individuals^2–9^, although he lacked some of the characteristic features such as the distinctive facial features and slender build. Individuals 2 and 3 exhibited intellectual disability with autistic features, but did not have cardiac symptoms; this is not surprising considering that a few reported patients with a *NONO* LoF variant did not have cardiac symptoms^2,7^. The *NONO* variant is suggested to result in a wide variety of expressivity because of mild LoF. The *NONO* variant may function as a modifier of as-yet-unidentified variants^18^.

Our results showed that the rough-eye phenotype could reflect mild LoF associated with missense variants. The *Drosophila* eye consists of 700–800 basic units called ommatidia. They are aligned in an orderly manner but become disordered with the infusion of ectopic genes that function within them^19^. Rough-eye phenotype can be performed without special equipment other than an epimicroscope. Most rare variants are missense variants^20^. Missense variants can be deleterious via three mechanisms: LoF, gain of function, or dominant negative effects^21^. It is difficult to determine which function a missense variant has using only in silico tools. In such cases, the rough-eye phenotype can be a good option. Based on our findings, we determined that the Pro459Ala variant resulted in a LoF due to the milder rough eye phenotype observed. However, it is difficult to completely rule out the potential for dominant-negative effects or weak gain-of-function that may not have been detected using the *Drosophila* experimental system. Further validation using human cultured cells or mammalian models may be needed in the future to fully understand the functional consequences of this variant.

The identified *NONO* variant was located in the NLS region and impaired proper nuclear localization. Proper localization of proteins is essential for their functions, and some studies have shown that the impairment of NLS can cause human diseases^22–24^. NLS consists of several motifs, but there are redundancies in each of them^25,26^; therefore, it is not straightforward to assess how the identified variant affects NLS. In our case, NONO protein with the variant could enter the nucleus, but its nuclear localization was diffuse. Unfortunately, we could not identify the cause of this.

In conclusion, we identified a novel missense variant in *NONO* that caused clinical phenotypes that overlapped with previously reported symptoms associated with *NONO* LoF variants. Deleterious missense variants in *NONO* should be considered when genetic testing is performed on male individuals with developmental delay with or without cardiac symptoms. Considering that *NONO* is intolerant of missense variants (missense Z-score in gnomAD: 3.59), it is plausible that other deleterious missense variants in functional regions or domains can cause human diseases. Future studies should focus on whether other deleterious missense variants in functional modules can cause similar symptoms.

## Supplementary Information

Supplementary Information.

## Data Availability

All data generated or analyzed in this study are included in this published article.
