# Publisher Correction: A novel *NONO* variant that causes developmental delay and cardiac phenotypes

**DOI:** 10.1038/s41598-023-30968-3

**Published:** 2023-03-09

**Authors:** Toshiyuki Itai, Atsushi Sugie, Yohei Nitta, Ryuto Maki, Takashi Suzuki, Yoichi Shinkai, Yoshihiro Watanabe, Yusuke Nakano, Kazushi Ichikawa, Nobuhiko Okamoto, Yasuhiro Utsuno, Eriko Koshimizu, Atsushi Fujita, Kohei Hamanaka, Yuri Uchiyama, Naomi Tsuchida, Noriko Miyake, Kazuharu Misawa, Takeshi Mizuguchi, Satoko Miyatake, Naomichi Matsumoto

**Affiliations:** 1 Department of Human Genetics, Yokohama City University Graduate School of Medicine, 3-9 Fukuura, Kanazawa-Ku, Yokohama, Kanagawa Japan; 2 Brain Research Institute, Niigata University, Niigata, Japan; 3 School of Life Science and Technology, Tokyo Institute of Technology, Yokohama, Kanagawa Japan; 4 Biomedical Research Institute, National Institute of Advanced Industrial Science and Technology (AIST), Tsukuba, Ibaraki Japan; 5 Children’s Medical Center, Yokohama City University Medical Center, Yokohama, Kanagawa Japan; 6 Department of Pediatric Cardiology, Yokohama City University Hospital, Yokohama, Kanagawa Japan; 7 Department of Pediatrics, Fujisawa City Hospital, Fujisawa, Kanagawa Japan; 8 Department of Medical Genetics, Osaka Women’s and Children’s Hospital, Izumi, Osaka Japan; 9 Department of Rare Disease Genomics, Yokohama City University Hospital, Yokohama, Kanagawa Japan; 10 Department of Human Genetics, Research Institute, National Center for Global Health and Medicine, Tokyo, Japan; 11 RIKEN Center for Advanced Intelligence Project, Tokyo, Japan; 12 Clinical Genetics Department, Yokohama City University Hospital, Yokohama, Kanagawa Japan

Correction to: *Scientific Reports* https://doi.org/10.1038/s41598-023-27770-6, published online 18 January 2023

In the original version of this Article Toshiyuki Itai and Atsushi Sugie were omitted as equally contributing authors.

In addition, in Table 1, a “–” sign was omitted for “Craniofacial/Neck features” of “Individual 3”.

Incorrect:

**Table:** 

	Family 1	Family 2	
	Individual 1	Individual 2	Individual 3
Craniofacial/Neck features	–	–	

Correct:

**Table:** 

	Family 1	Family 2	
	Individual 1	Individual 2	Individual 3
Craniofacial/Neck features	–	–	–

The original article has been corrected.

