# Supplementary material for: A novel NONO variant that causes developmental delay and cardiac phenotypes
Source: Sci Rep. 2023 Jan 18;13:975. doi: 10.1038/s41598-023-27770-6 (PMC9849200; doi:10.1038/s41598-023-27770-6)
Supplement: Supplementary file 1 — Supplementary Information. [file 41598_2023_27770_MOESM1_ESM.docx]

Supporting Information

A novel *NONO* variant that causes developmental delay and cardiac phenotypes.

**Contents**

Supplementary figure

Figure S1. Original images of western blotting in Fig. 2a p. 1

**Figure S1. Original images of western blotting in Fig. 2a**

**
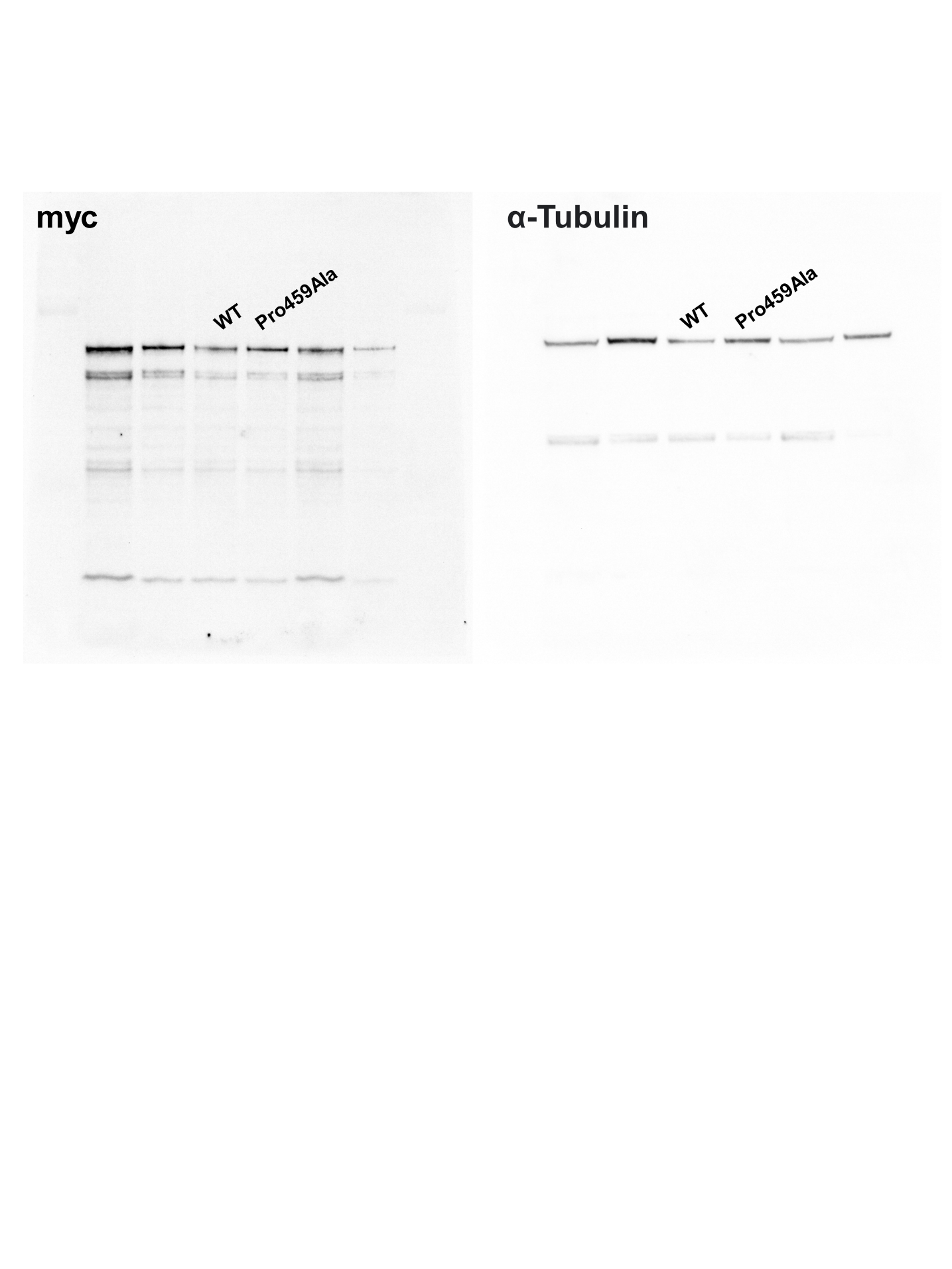
**

The upper row of the third and fourth lanes from left were used in Fig. 2a in the manuscript.
